# Poloxamer-based injectable hydrogels as matrices for localized anti-inflammatory drug delivery in meniscus injuries

**DOI:** 10.1039/d6ra02085b

**Published:** 2026-05-01

**Authors:** Marta Tuszynska, Adriana Gonçalves, Joanna Skopinska-Wisniewska, Paula I. P. Soares, Anna Bajek

**Affiliations:** a Department of Oncology, Ludwik Rydygier Collegium Medicum in Bydgoszcz Nicolaus Copernicus University in Torun Lukasiewicza 1 St. 85-821 Bydgoszcz Poland 503348@doktorant.umk.pl marta.tuszynska96@gmail.com; b i3N/CENIMAT, Department of Materials Science, NOVA School of Science and Technology, NOVA University Lisbon Campus de Caparica 2829-516 Caparica Portugal; c Laboratory for Functional Polymeric Materials, Faculty of Chemistry, Nicolaus Copernicus University in Torun Gagarina 7 St. 87-100 Torun Poland

## Abstract

Poloxamer-based hydrogels, composed of thermoreversible triblock copolymers, are promising drug delivery systems due to their ability to transition from a liquid to a gel state at physiological temperatures, enabling minimally invasive injection and localized, sustained release of therapeutic agents. In this study, poloxamer hydrogels were prepared with diclofenac sodium salt and paracetamol as model anti-inflammatory drugs, and characterized for morphology, osmolarity, pH, and temperature sensitivity. Drug loading optimization was performed to ensure homogeneous dispersion, and release kinetics were evaluated by spectrophotometric analysis, with mathematical modeling used to describe and predict drug release mechanisms from the hydrogel matrix. The optimized poloxamer gels exhibited an appropriate sol–gel transition near body temperature (26–37 °C), stable pH, and osmolarity suitable for biomedical use. Drug release profiles showed controlled, sustained release of both diclofenac sodium and paracetamol over extended periods, with mathematical modeling indicating that diffusion-based mechanisms predominated in drug release from the hydrogel matrix, validating the system design for targeted, localized therapy. These findings demonstrate that poloxamer-based injectable hydrogels effectively deliver anti-inflammatory agents with controlled release, representing a versatile platform for localized drug delivery in regenerative medicine and orthopedic applications, particularly for intra-articular treatment of musculoskeletal disorders, thereby supporting improved therapeutic outcomes while minimizing systemic exposure and associated side effects.

## Abstract 2

Poloxamer-based hydrogels are a promising matrix for controlled injectable drug delivery, exhibiting a physiological sol–gel transition, stable pH and osmolarity, and sustained release of diclofenac and paracetamol for osteoarticular injuries.

## Graphical abstract

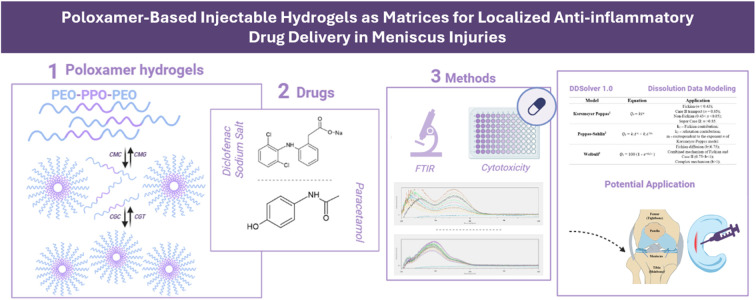

## 1. Introduction

Drug delivery systems are designed to control the release and distribution of therapeutic agents, improving therapeutic efficacy while minimizing systemic side effects.^1,2^ Hydrogel matrices have emerged as particularly promising carriers due to their hydrophilic, crosslinked polymer networks, which can hold large volumes of water while maintaining structural integrity.^3,4^ Their softness, porosity, high biocompatibility, and stimuli-responsive behavior, such as sol–gel transitions triggered by temperature, pH, or enzymatic activity, enable precise control of drug release *in vivo*. Thermosensitive hydrogels have attracted significant attention due to their ability to undergo phase transitions as ambient temperature changes. This property enhances drug delivery systems by improving local drug penetration, providing better spatial and temporal control, and increasing drug bioavailability.^4,5^

Poloxamer-based hydrogels, composed of nonionic triblock copolymers of poly(ethylene oxide)–poly(propylene oxide)–poly(ethylene oxide) (PEO–PPO–PEO), exhibit thermoreversible gelation behavior, remaining liquid at room temperature and transitioning to a gel at physiological temperatures.^6,7^ These polymers, known commercially as Pluronics, Synperonics, or Lutrol, include a variety of liquids, pastes, and solids. Their molecular weights range from 1100 to 14 000, and the ethylene oxide to propylene oxide weight ratios vary from 1 : 9 to 8 : 2.^8^ Aqueous poloxamer 407 undergoes a solution-to-gel transition as temperature increases, caused by the formation and organization of micelles into periodic lattices.^9^ This property allows for minimally invasive administration *via* injection, followed by *in situ* gelation at the target site.^10,11^ Recent advances have addressed the inherent limitations of poloxamer hydrogels, including weak mechanical properties and rapid gel erosion, by employing chemical crosslinking strategies that extend drug release profiles to 70 days while maintaining biocompatibility. The nature, composition, and concentration of poloxamers are the most critical factors defining drug release rates, with hydrophobic gel matrices exhibiting compact micellar arrangements that slow diffusion and erosion.^12^ Incorporating drug-loaded particles into poloxamer gels extends drug release by establishing multiple barriers that limit the release rate. Meanwhile, chemical modification of poloxamers offers a promising approach to achieve prolonged sustained release for parenteral use, without sacrificing the gel's rheological properties.^1^

Hydrophobic small molecules can be encapsulated inside poloxamer matrices, but these additives often adversely affect the rheological properties and reduce the gelation temperatures of the hydrogels, which may limit their clinical use. Studies employing differential scanning calorimetry, rheology, and small-angle X-ray scattering have demonstrated that small molecule addition lowers thermal transition temperatures and increases micelle size, providing fundamental understanding for tuning the mechanical and structural properties of drug-loaded formulations.^13^ As demonstrated in studies on poloxamer formulations, increasing gelling viscosity reduces the drug release rate and gel dissolution time, thereby prolonging the drug's duration of action in disease treatment.

In orthopedic and musculoskeletal applications, injectable hydrogels have demonstrated significant therapeutic potential as delivery vehicles for diverse bioactive agents, including growth factors, anti-inflammatory drugs, steroids, and cells.^14^ The meniscus, a fibrocartilaginous structure with limited intrinsic healing capacity due to its avascular nature, particularly benefits from localized delivery of therapeutic agents.^11^ Recent studies have validated the use of anti-inflammatory agents in thermosensitive hydrogels for intra-articular applications, demonstrating regenerated cartilage profiles and superior anti-inflammatory effects in osteoarthritis models. Injectable hydrogel drug delivery platforms offer many benefits for osteoarthritis treatment, including enhanced biocompatibility, biodegradability, and low immunogenicity, with mechanisms such as anti-inflammation, anti-oxidative stress, and promotion of cartilage regeneration.^15,16^ The concentration and composition of poloxamers greatly influence their biomedical applications. Triblock copolymer systems offer desirable features for surgical use, including rapid sol–gel transition and biocompatibility, indicating their potential for osteoarticular regeneration.^17^

**Fig. 1:**
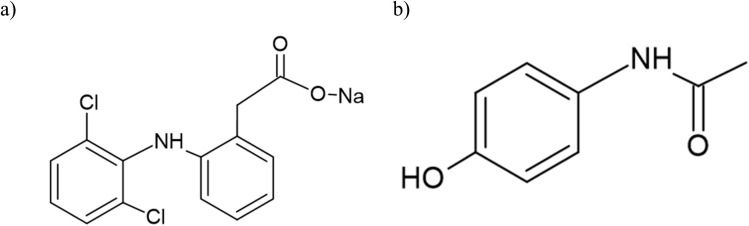
(a) Chemical structure of diclofenac sodium salt, (b) chemical structure of paracetamol.

Diclofenac sodium salt (Fig. 1a), a phenylacetic acid derivative, exerts its therapeutic effects primarily by inhibiting cyclooxygenase enzymes (COX-1 and COX-2), thereby preventing the conversion of arachidonic acid into prostaglandins.^18–20^ Paracetamol (*N*-acetyl-*p*-aminophenol) (Fig. 1b) is among the most extensively utilized agents with antipyretic, analgesic, and anti-inflammatory properties.^21,22^ Poloxamer formulations enhance the solubility of both agents and enable sustained release profiles. Combining these agents with poloxamer-based hydrogel systems allows localized, sustained, and controlled release at the injury site, enhancing tissue integration and reducing systemic exposure.^5,23^

Mathematical modeling is essential for analyzing and predicting drug release behavior from hydrogel matrices.^16,24^ The field originated with Higuchi's seminal model in 1961, and models have been categorized as empirical/semi-empirical or mechanistic. Mechanistic models, based on physical and chemical processes such as diffusion, dissolution, swelling, and degradation, offer deeper insights and better predictive power for optimizing system design. Recent studies confirm that diffusion-based mechanisms predominate in drug release from poloxamer matrices, with gel erosion and polymer dissolution ultimately controlling release kinetics.^25–29^

This study aimed to prepare poloxamer-based hydrogel matrices loaded with diclofenac sodium salt and paracetamol, as model anti-inflammatory drugs, with a primary focus on evaluating controlled-release behavior and functional performance. The obtained drug-loaded gels were characterized for morphology, osmolarity, pH, temperature sensitivity, and drug loading to optimize drug dispersion and release properties. Release kinetics were assessed by spectrophotometric analysis, and mathematical models were applied to describe and predict drug release mechanisms from the poloxamer-based hydrogel matrix.

## 2. Materials and methods

### 2.1 Materials

Kolliphor® K407 (K407), Poloxamer 188 Pro (P188), and Synperonic® F108 (F108) were purchased from MERCK Sigma-Aldrich (St. Louis, MO, USA). *N*-(4-Hydroxyphenyl)acetamide (paracetamol) and diclofenac sodium salt were kindly offered by the NOVA FCT University (MERCK Sigma-Aldrich). All chemicals were used as received without additional purification. Ultra-pure water was produced in-house using a Milli-Q water purification system (Millipore, Merck, Darmstadt, Germany).

### 2.2 Preparation of poloxamer-based hydrogels

Previously selected groups of hydrogels were prepared following final concentration formulations: F108_P188 10%_20% (FP 1020); F108_P188 15%_15% (FP 1515); K407_P188 20%_10% (KP 1020); K407_P188 15%_15% (KP 1515), Table 1. Formulations were prepared by mechanical dispersion in phosphate buffer solution with pH 7.4 (PBS 7.4) at 4 °C and refrigerated for 24 h before further analysis.

**Table 1:** Specification of name and concentration of described poloxamer-based hydrogels

Kolliphor 407 [wt%]	Synperonic F108 [wt%]	Poloxamer 188 [wt%]	Name	Paracetamol [wt%]	Diclofenac sodium salt [wt%]
K	F	P	Polymer mix [%, %]	PARAC	DICLO
10	—	20	**K**P 1020	0.1/0.5/1	0.5/1
15	—	15	**K**P 1515	0.1/0.5/1	0.5/1
—	10	20	**F**P 1020	0.1/0.5/1	0.5/1
—	15	15	**F**P 1515	0.1/0.5/1	0.5/1

#### 2.2.1 Preparation of polymeric drug-loaded hydrogels

The hydrogels were prepared using the procedure described previously.^7^ The poloxamer mixture was continuously stirred at 250–300 rpm at 4 °C until a clear solution was obtained. Subsequently, the samples were stored overnight at 4 °C.

(1) Different concentrations of PARAC (0.1%, 0.5% and 1% w/v) or DICLO (0.5% and 1% w/v) were added to the obtained poloxamer solutions and stirred at 300 rpm in an ice bath until the solution became clear. Then, the samples were refrigerated overnight at 4 °C.

(2) Weighted powdered form of PARAC (0.1%, 0.5% and 1% w/v) or DICLO (0.5% and 1% w/v) in different concentrations was added to the obtained solutions and mixed with poloxamer-gel at 37 °C until the solution became clear. Then, the samples were placed in dialysis bags to stabilize for 20 min at 37 °C.

### 2.3 Osmolarity, pH

Osmolarity of drug-loaded and basic poloxamer gel solutions was analyzed using a KNAUER Freezing Point Osmometer K-7400S. This osmometer measures from 0 to 2000 mOsmol per kg with a resolution of 1 mOsmol per kg. Calibration was carried out with water (Carlo Erba Reagents, HPLC plus) as 0 mOsmol per kg and standard solutions of 400 and 850 mOsmol per kg. The reported values are the average of three measurements.

The pH of the pre-gels was measured at room temperature using a pH meter (Mettler Toledo, Toledo, OH, USA). All measurements were taken at 22–25 °C. Components were dissolved in PBS in a beaker, and the electrode was immersed for testing. The values shown are averages of three measurements.

### 2.4 Cytotoxicity assay

Cell viability studies were conducted on the Vero fibroblast-like cell line using the resazurin assay. Cells were seeded at a density of 5 × 10^4^ cells per ml in 96-well plates. The Vero cell line was cultured in Dulbecco's modified Eagle's medium (DMEM) supplemented with 10% fetal bovine serum, 1% Penicillin-Streptomycin (10 000 U ml^−1^), sodium pyruvate (100 mM), and GlutaMAX™ Supplement, then incubated at 37 °C in 5% CO_2_ overnight. The next day, cells were treated with fresh medium containing known concentrations of hydrogel extracts (0.1%, 1%, 50%, and 100%) in triplicate, and the plates were incubated for 24, 48, and 72 hours. After incubation, the medium was removed, and resazurin was added to each well. Following 2 hours of incubation, absorbance was measured at 570 and 600 nm. Control cells underwent the same procedure and were incubated in the respective medium at the same dilutions as those used for the hydrogel extracts. Cell viability was expressed as a percentage of the control, calculated as [% cell viability = (extracts treated cells/control cells) × 100].

### 2.5 Drug release calibration curves

Calibration curves of controls were made by dialyzing 1 ml of free drug in the release medium, PBS 7.4. Drug calibration curves were constructed to determine PARAC and DICLO concentration through further assays by measuring the absorbance using a UV-Vis spectrophotometer (UV-Vis ScanSci Sarspec, SPEC RES+ Instruments). The integration time was set to 145 seconds. Measurement was made in concentrations between 1 and 12 µg ml^−1^ of paracetamol and between 1 and 35 µg ml^−1^ of diclofenac sodium salt. The maximum absorbance peak at 242 nm and 275 nm for paracetamol and diclofenac, respectively, was determined at different drug concentrations. The visualization was made using the LighScan. These measures to obtain the calibration curves were made in triplicate.

#### 2.5.1 *In vitro* drug release

An *in vitro* hydrogel release study was carried out as follows: 1 ml of poloxamer-drug hydrogel was added to a dialysis bag 12–14 KDa, sealed on both sides, and then placed into a pre-thermostated PBS 7.4 15 ml solution in a 50 ml Falcon. The release profile was carried out and maintained at 37 ± 2 °C. Periodically, 2 ml of release medium was removed and replaced with an equal volume of new buffer. The absorbance of all samples was measured at 242 nm for PARAC and at 275 nm for DICLO. These absorbance values, in combination with calibration curves for paracetamol and diclofenac, allowed the determination of drug concentration and the calculation of the percentage of released drug over time.

#### 2.5.2 Drug release mechanisms

The cumulative amounts of diclofenac and paracetamol *in vitro* were plotted at the graphical interface using several kinetic models, including Korsmeyer–Peppas, Peppas–Sahlin, and Weibull, to characterize the drug release mechanism (Table 2). The model with the highest *R*^2^ (regression coefficient) was considered the best mechanism for representing the kinetic release. The exponent (*n*) determined the release mechanism, where Fickian diffusion represents *n* values of 0.5 or near it, and non-Fickian diffusion represents *n* values of 1.0 or near it.

**Table 2:** Summary of the used mathematical models for drug delivery systems

Model	Equation	Application	Ref.
Korsmeyer–Peppas^a^	*Q*_*t*_ = *kt*^*n*^	Fickian (*n* ≤ 0.43)	26, 30 and 31
		Case II transport (*n* = 0.85)	
		Non-Fickian (0.43 < *n* < 0.85)	
		Super Case II: *n* > 0.85	
Peppas–Sahlin^b^	*Q*_*t*_ = *k*_1_*t*^*m*^ + *k*_2_*t*^2*m*^	*k*_1_ – Fickian contribution	
		*k*_2_ – Relaxation contribution	
		*m* – Correspondent to the exponent *n* of Korsmeyer–Peppas model	
Weibull^c^	*Q*_*t*_ = 100 (1 − e^−*tb*/*a*^)	Fickian diffusion (*b* ≤ 0.75)	
		Combined mechanism of Fickian and Case II (0.75 < *b* < 1)	
		Complex mechanism (*b* > 1)	

a *n*, Empirical release exponent.

b *k*, Related to polymer structure and geometry.

c *Q*_*t*_, Amount of drug released at time *t*.

### 2.6 Statistical analysis

Statistical analysis was performed using GraphPad Prism software. Statistical differences were determined by analysis of variance (ANOVA). Numerical and graphical results are displayed as mean ± standard deviation. Significance was accepted at a level of *p* < 0.05.

## 3. Results and discussion

### 3.1 Hydrogel selection and characterization

Previously presented results summarized the compositions of many formulations tested in this work.^7^ Following thorough formulation analysis, only two formulations were selected for their effective gelling properties. Specifically, these materials were chosen due to their ability to form a gel within the target temperature range of 35 to 37 °C, mimicking the physiological conditions of the meniscus surface.^32^ This temperature range is crucial for injectable hydrogel applications. Additionally, a quick gelation time is necessary for medical use to provide a sufficient waiting period before achieving the desired consistency. The formulations' fluidity at room temperature offers another benefit, as it enables easy sterilization *via* filtration in their liquid form, which is essential for commercial applications.^33^ Injectable hydrogels have emerged as promising options for osteoarthritis treatment due to their ability to deliver bioactive molecules directly to the affected joint, enhancing local effectiveness while reducing systemic side effects.

### 3.2 Physical appearance of preparation

**Fig. 2:**
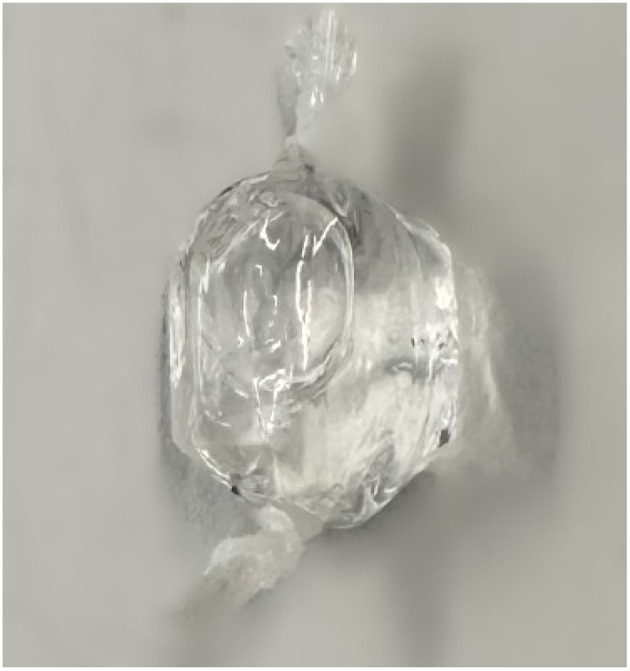
The visual appearance of the hydrogel sample mixed with the drug in the dialysis bag.

The physical appearance was inspected visually. The appearance of the poloxamer-based hydrogel single and with the addition of the tested drug appeared as a clear, homogeneous, and liquid solution at 4 °C up to room temperature (Fig. 2). This observation is consistent with recent studies demonstrating that low percentages of drug incorporated into poloxamer hydrogels do not significantly affect their visual and textural characteristics. The homogeneous appearance indicates successful dispersion of the drug within the hydrogel matrix, which is essential for uniform drug release profiles.

### 3.3 Osmolarity, pH

The meniscus surface is in a constant dynamic of production, evaporation, and absorption.^34–36^ The systems from Table 3 were all measured by freezing point osmometry, and all hydrogel formulations presented osmolalities within the physiological values of around 300 mOsmol per kg.^37^ The extrapolated osmolarity value for the undiluted gel was around 287 ± 2 mOsmol per kg. The pH of the pre-gels was approximately 7.4 ± 2 in all cases, which falls within the tolerable range for osteoarticular delivery. The average osmolarity of the freshly prepared gels was consistent across all samples, indicating that osmolarity remained unaffected by poloxamer concentration.

**Table 3:** Osmolality, pH values of PARAC and DICLO samples in poloxamer-hydrogels. Data indicated as mean + SD

Sample		Osmolality [mOsm per kg]	pH in PBS
KP1515		295 ± 2.65	7.47
DICLO	1%	287 ± 2.52	7.46
	0.5%	285 ± 1.00	7.48
PARAC	1%	293 ± 2.65	7.50
	0.5%	289 ± 0.00	7.49
	0.1%	293 ± 1.00	7.51
FP1020		299 ± 0.58	7.48
DICLO	1%	297 ± 1.73	7.50
	0.5%	294 ± 0.58	7.49
PARAC	1%	292 ± 1.15	7.44
	0.5%	292 ± 0.58	7.46
	0.1%	295 ± 1.73	7.48

The results confirm that the physiological parameters are maintained in all tested samples. We compared samples and found that the lowest osmolality was 285 mOsmol per kg, while the highest was 299 mOsmol per kg, indicating no significant difference in osmolarity among the tested materials. Generally, the addition of the drug did not affect the osmolarity of the hydrogel matrix, consistent with previous findings that the chemical nature of drugs incorporated into poloxamer hydrogels influences the pH of preparations, while low drug incorporation levels do not significantly affect other physicochemical characteristics.^38–40^ Furthermore, none of the samples significantly altered the pH of a phosphate-buffered solution, indicating they do not affect physiological pH levels. This finding is particularly relevant for intra-articular applications, where maintaining physiological pH is essential to avoid tissue irritation and ensure biocompatibility.^5,41^ This study suggests that these osmolarity values are not a limitation to the use of paracetamol or diclofenac in a future formulation.

Recent investigations have demonstrated that acetaminophen (paracetamol) loading affects the physical and structural properties of poloxamer 407 micelles and hydrogels. Differential scanning calorimetry showed that acetaminophen lowers both the critical micelle temperature and the enthalpy of micellization in poloxamer solutions, promoting micellization.^42^ However, at the concentrations used in this study (0.1–1%), the drug loading did not substantially alter the osmolarity or pH of the formulations, supporting their suitability for biomedical applications. A mechanistic understanding of the changes in the physical properties of poloxamer gels induced by drug loading is essential for the effective development of poloxamer gel formulations.

### 3.4 Cytotoxicity

**Fig. 3:**
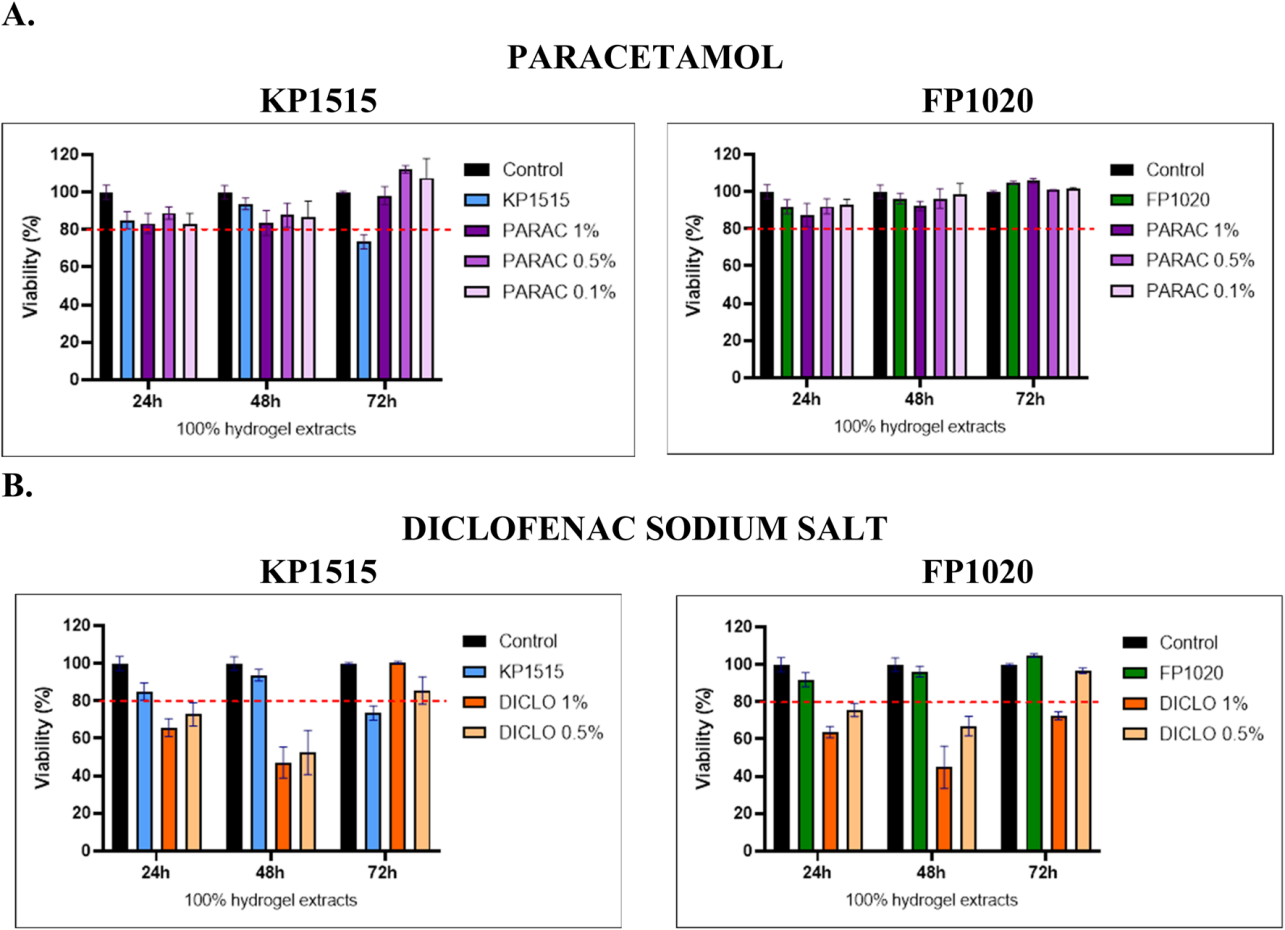
Relative cell viability after 24, 48, and 72 h of incubation with hydrogel extracts supplemented with (A) paracetamol and (B) diclofenac sodium salt. The dashed line indicates the minimum percentage of cell viability above which extracts are not considered cytotoxic.

One of the most essential characteristics of a biomaterial is its toxicity. In the present study, poloxamer-based hydrogels loaded with PARAC or DICLO were evaluated for cytotoxicity *in vitro* using the Vero cell line. Fig. 3 presents the Vero cell line viability in response to exposure to hydrogel-drug-loaded extracts after 24 h, 48 h, and 72 h of incubation. This study aimed to evaluate the biocompatibility of the tested compositions for use in meniscus regeneration. The effect of extract contact with the cell line was assessed using a resazurin assay to evaluate cell viability.

The resazurin assay performed on Vero cells indicates that all paracetamol-loaded formulations (PARAC) present high biocompatibility across the tested concentration range. On the other side, 1% and 0.5% diclofenac-based hydrogel (DICLO) in the first hours do not reach the minimum viability percentage. Viability is modestly reduced after 48 h, suggesting that early incubation is the most critical for cell stress, whereas 72 h exposures generally support cell proliferation (Fig. 1). This delayed recovery is consistent with the known capacity of hydrogel matrices to provide a permissive microenvironment for cell attachment and growth, as supramolecular hydrogels have been shown to promote biocompatibility and controlled release of bioactive agents. Hydrogels containing PARAC display higher biocompatibility than DICLO at 24 h, likely because the paracetamol-loaded network modulates the local pH more gently. Ion-exchange reactions at the hydrogel-medium interface can raise pH during the first 24 h, a phenomenon previously linked to increased cytotoxicity in ionically cross-linked systems. In contrast, the KP1515 formulation shows a progressive decline in viability with more prolonged exposure, which may reflect a faster dissolution rate and a more pronounced pH shift that impairs cellular metabolism. When hydrogel FP1020 is combined with PARAC, cell viability remains above the ISO 10993-accepted threshold (≥80% relative to control) throughout the experiment, indicating stable cytotoxicity profiles. Adding DICLO to FP1020 produces an initial dip at 48 h, followed by a rebound above the norm at 72 h, suggesting that cells adapt to the transient pH change and resume proliferation. Overall, at the concentrations examined, the hydrogel extracts do not pose a sustained toxic threat; *in vivo* pH-buffering mechanisms are expected to mitigate further any temporary acidity or alkalinity spikes.^26,31,43^

### 3.5 Drug release

#### 3.5.1 Calibration curves

**Fig. 4:**
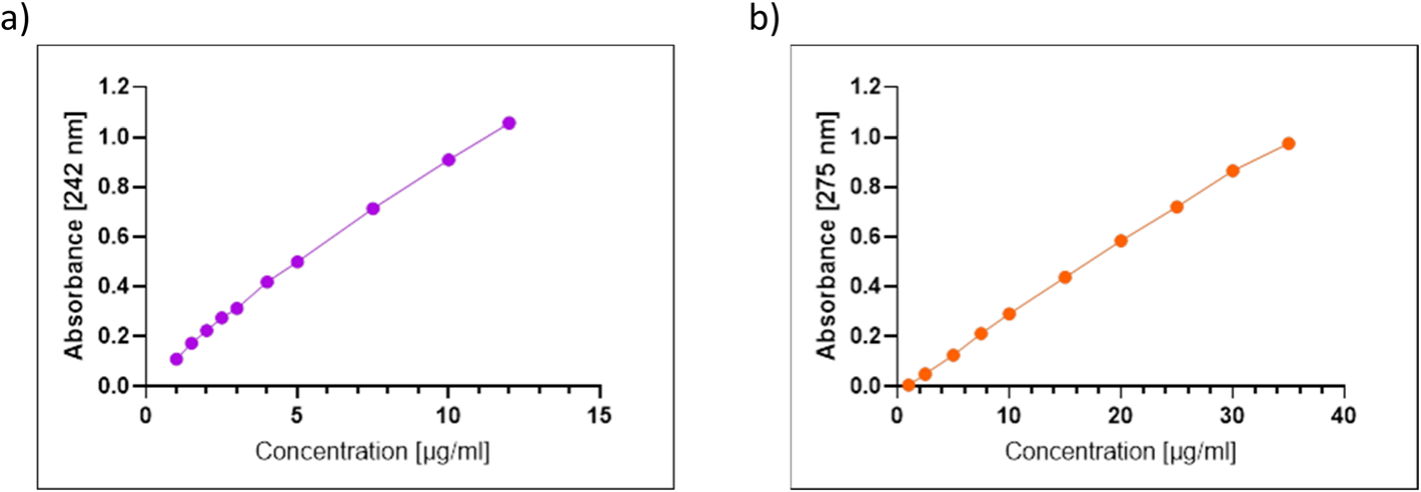
(a) Calibration curve of paracetamol in PBS (pH 7.4), the linear line over concentration ranged from 1 to 12 µg ml^−1^. The regression coefficient (*R*^2^) was 0.9971, (b) calibration curve of diclofenac sodium salt in PBS (pH 7.4), the linear line over concentration ranged from 1 to 35 µg ml^−1^. The regression coefficient (*R*^2^) was 0.9983.

The calibration curve was generated by linear regression of analyte concentration *versus* absorbance, as shown in Fig. 4. For paracetamol-loaded poloxamer gels, the linear line was created over the concentration range 1 to 12 µg ml^−1^. The concentration range of 1–35 µg ml^−1^ was measured for diclofenac-loaded hydrogels. The regression coefficients (*R*^2^) were 0.9971 and 0.9983, respectively, for PARAC and DICLO. These results corresponded to the literature-reported findings.^22,28,44–47^ These absorbance values, in conjunction with drug calibration curves, enabled the determination of drug concentration and the calculation of the percentage of released drug over a specified period.

#### 3.5.2 *In vitro* drug release studies

Micellization, in definition, is a mechanism where micelles are formed.^48^ The self-assembly and gelation of poloxamers in aqueous solutions proceed *via* a two-step mechanism. In the initial phase, amphiphilic triblock copolymers spontaneously associate to form spherical micelles. These micelles are characterized by a hydrophilic corona composed of hydrated and expanded PEO chains, surrounding a hydrophobic core formed by dehydrated PPO segments.^7,49^ This micellization process is thermoresponsive and occurs upon heating the system to the critical micellization temperature (CMT). Upon further temperature increase, the micelles overlap and align in a more ordered direction, forming a three-dimensional gel network. This sol–gel transition is thermoreversible, such that cooling the system back to ambient temperature leads to the disassembly of the network and reversion to a viscous solution. It is well-established that the minimum concentration required for gelation decreases with increasing copolymer molecular weight and PPO block content.

Due to the amphiphilic nature of the poloxamer hydrogels, the release profile was tested at two temperatures to assess drug incorporation within the hydrogel matrix. Since the sol–gel transition occurs around body temperature, the first option, the selected drug concentrations were weighed and added directly to the dissolved hydrogel in the liquid phase at 4 °C, and the mixture was left to dissolve properly overnight. On the other hand, another sample was thermostated, and before reaching the gel state, the drug in a powdered form was added to the hydrogel matrix and mixed to obtain a clear solution. Both PARAC and DICLO dissolved perfectly in poloxamer-based gels. This confirms the increased solubility of the drugs due to the poloxamer structure. This also leads to these two dissolution options, because during the transition from sol to gel state, the poloxamer gels undergo micellization.

**Fig. 5:**
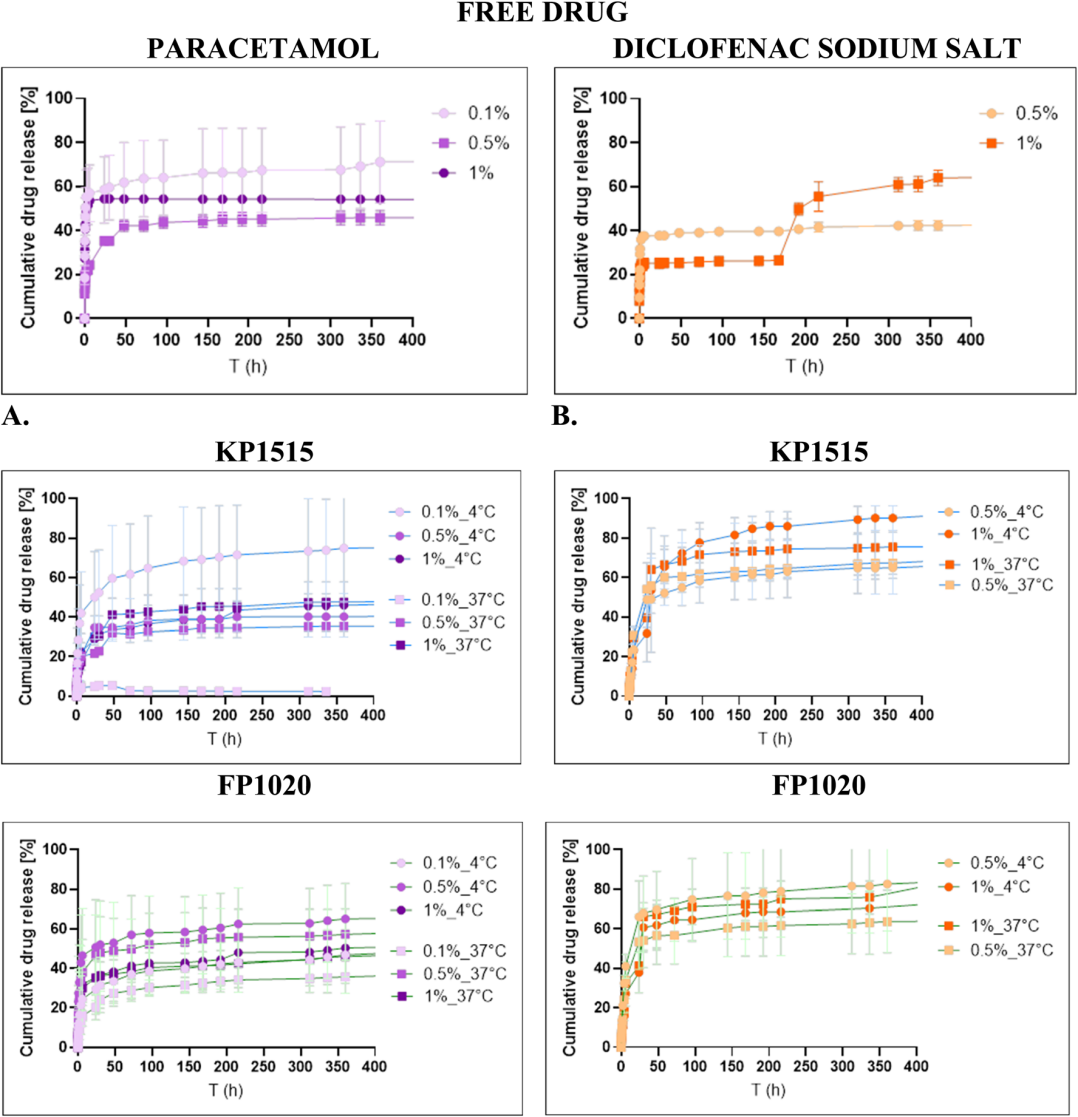
(A) Paracetamol and (B) diclofenac sodium salt release profile in different hydrogel matrices. The results are expressed as average ± standard deviation for three independent experiments.

The influence of polymer molecular weight (KP1515 and FP1020), drug type (PARAC *versus* DICLO), and formulation temperature (4 °C and 37 °C) on drug release kinetics was systematically investigated. A general similarity in the overall trend of the release profile was observed across all drug-loaded hydrogel formulations (Fig. 5).

Specific analysis revealed notable distinctions dependent on the drug type. For PARAC, the lowest cumulative release occurred at 0.1% loading at 37 °C, whereas the highest release was recorded for the same concentration at 4 °C. Interestingly, the release profile of the unbound (free) PARAC exceeded that of the loaded hydrogels. This contrasts with the behavior observed for DICLO, where the free drug exhibited minimal release. DICLO-loaded materials generally showed higher release profiles than PARAC formulations, with the maximum observed release occurring for 1% DICLO at 4 °C within the KP1515 matrix. Furthermore, the release profiles for materials loaded at 0.5% and 1% concentrations displayed kinetic similarity across various conditions, despite quantitative differences in the total released drug. Notably, KP1515 loaded with 0.1% PARAC showed negligible release at 37 °C.

These observed differences in release behavior are likely correlated with the temperature-dependent structural organization of the drug-hydrogel matrix. The initial formation conditions, particularly the temperature, dictate the structural state of the polymer network, such as micelle formation or crosslinking, which subsequently governs the drug retention mechanism, as previously established for polymer–drug systems in the literature.^43^ Temperature variations significantly impact the micellization process and the degree of hydration within the micellar core or hydrogel network, which modulates drug diffusion and release rates.^50,51^ For example, in poloxamer systems, temperature changes affect micelle packing and physical properties like gel strength.^42^ Moreover, the solubilization and structural properties of Pluronic micelles are sensitive to temperature, favoring micelle formation at lower temperatures through increased hydration of the PEO corona.^50^

#### 3.5.3 Drug release mechanisms

Selecting the correct mathematical model is crucial for accurately fitting experimental drug release data. When utilizing drug carriers like poloxamer hydrogels, which function as swelling matrices, conventional models such as zero-order, first-order, Higuchi, Hixson–Crowell, and Hopfenberg are often inappropriate. Zero-order kinetics is suited for drugs with low solubility in non-swelling matrices, while first-order kinetics describes dissolution from porous materials where water solubility is high. The Higuchi model, derived for non-swelling matrices, is unsuitable because poloxamer hydrogels swell upon hydration.^30^ Furthermore, Hixson–Crowell assumes constant surface area, and Hopfenberg models assume polymer erosion, neither of which applies to typical poloxamer gel behavior under these conditions.^30,52^

Consequently, the Korsmeyer–Peppas, Weibull, or Peppas–Sahlin models often best describe the release kinetics from such polymer systems, which account for relaxation and swelling mechanisms. Model appropriateness is determined by selecting the equation that yields the highest adjusted coefficient of determination (*R*_adj_^2^),^53^ calculated as:
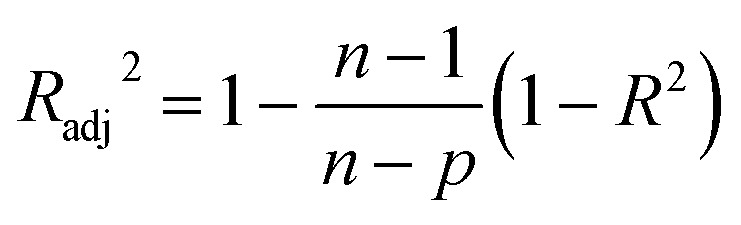


Considering the macroscopic geometry, the hydrogel's irregular shape, the *n* values were assumed. The Peppas–Sahlin model separates Fickian diffusion from relaxational contributions using constants *k*_1_ and *k*_2_.^54^ The exponent *n* from the Korsmeyer–Peppas model reveals the mechanism: *n* ≤ 0.43 indicates Fickian diffusion, while *n* = 0.85 signifies Case II transport driven by polymer relaxation.^26^ The Weibull model provides correlation *via* shape factor *b*, where *b* ≤ 0.75 suggests Fickian diffusion and *b* > 1 implies a complex release mechanism.^55^

For the application of the chosen mathematical model, poloxamers at room and lower temperatures are considered to be in a liquid state. As such, fitting was performed for samples closed in the membrane to retain all the tested material. Korsmeyer–Peppas, Weibull, and Peppas–Sahlin models were applied to experimental data of DICLO and PARAC release from poloxamer-based hydrogels, at 4 °C and 37 °C. These two cases were tested to consider micelle creation. The results obtained from the DDSolver program^53^ are represented in Table 4. Parameter values of each mathematical model and *R*_adj_^2^ are described. Moreover, the higher *R*_adj_^2^ of each sample is highlighted in bold and yellow.

**Table 4:** Correlation coefficient *R*^2^ of different release models

Sample		Korsmeyer–Peppas			Weibull			Peppas–Sahlin			
		*n*	*K*	*R*_adj_^2^	*α*	*β*	*R*_adj_^2^	*k*_1_	*k*_2_	*m*	*R*_adj_^2^
Free PARAC	1%	0.350	50.880	**0.999**	1.520	0.394	**0.999**	67.661	−20.356	0.422	**0.999**
	0.5%	0.179	19.860	0.979	4.399	0.169	0.988	30.302	−9.525	0.327	**0.994**
	0.1%	0.273	51.181	0.926	1.387	0.294	0.976	93.390	−37.900	0.564	**0.993**
KP1515	1%_4 °C	0.372	11.413	0.968	8.397	0.349	0.983	14.541	−2.762	0.492	**0.995**
	1%_37 °C	0.331	11.076	0.937	8.662	0.308	0.960	16.359	−4.063	0.595	**0.982**
	0.5%_4 °C	0.308	15.232	0.902	5.681	0.278	0.937	23.914	−6.580	0.591	**0.977**
PARAC	0.5%_37 °C	0.341	13.833	0.922	6.319	0.310	0.955	20.862	−5.330	0.607	**0.978**
	0.1%_4 °C	0.529	23.185	0.938	4.622	0.525	0.971	18.936	7.260	0.572	**0.987**
	0.1%_37 °C	0.492	2.147	**0.960**	47.567	0.494	0.939	1.157	1.100	0.371	0.943
FP1020	1%_4 °C	0.384	19.882	0.943	4.554	0.372	0.971	27.388	−5.463	0.619	**0.982**
	1%_37 °C	0.443	17.255	0.959	6.381	0.440	0.975	22.049	−3.566	0.638	**0.977**
	0.5%_4 °C	0.261	26.915	0.828	3.791	0.268	0.927	33.084	−4.631	0.510	**0.944**
PARAC	0.5%_37 °C	0.301	20.818	0.867	4.660	0.340	0.913	23.714	−2.553	0.568	**0.970**
	0.1%_4 °C	0.557	13.370	0.946	8.531	0.498	0.973	−2.260	17.377	0.335	**0.986**
	0.1%_37 °C	0.619	7.187	0.968	11.566	0.526	0.989	−7.783	15.637	0.383	**0.990**
Free DICLO	1%	0.086	19.153	0.786	4.515	0.085	0.865	38.850	−13.831	0.600	**0.998**
	0.5%	0.668	36.394	0.976	2.848	0.326	**0.999**	5821.41	5852.11	0.002	0.989
KP1515	1%_4 °C	0.538	10.058	0.914	10.872	0.672	0.915	8.603	0.148	0.674	**0.921**
	1%_37 °C	0.677	11.734	0.995	8.501	0.784	0.993	1.715	10.038	0.420	**0.996**
DICLO	0.5%_4 °C	0.827	5.410	0.998	17.261	0.854	**0.999**	−2.710	8.217	0.354	**0.999**
	0.5%_37 °C	0.677	8.423	**0.989**	13.259	0.551	**0.989**	−71.589	78.894	0.384	**0.989**
FP1020	1%_4 °C	0.717	9.899	0.998	10.050	0.802	0.996	3.406	6.385	0.434	**0.999**
	1%_37 °C	0.687	11.259	**0.999**	8.696	0.855	0.995	5.831	5.348	0.521	0.998
DICLO	0.5%_4 °C	0.620	13.529	**0.999**	6.840	0.709	0.998	0.819	12.791	0.347	**0.999**
	0.5%_37 °C	0.693	9.290	0.989	11.476	0.829	0.984	5.651	3.505	0.478	**0.991**

Almost all experimental data are well fitted by the Peppas–Sahlin model, although slight deviations are found from the Korsmeyer–Peppas and Weibull adjusted coefficient of determination. Parameters from the other two models were also analyzed to understand these differences. Exponent *n* from the Korsmeyer–Peppas model presents a value below 0.85 for all cases, indicating an anomalous or non-Fickian diffusion. The values are much lower for the samples loaded with PARAC compared to DICLO, where the *n* value approaches 0.6, which is close to the established limit between Fickian and non-Fickian diffusion.^56,57^ On the other hand, exponent *m* from the Peppas–Sahlin model has an average value of 0.47 for all cases, which indicates a Fickian diffusion mechanism of release. The value of the drug release exponent from the hydrogel containing diclofenac is above 0.5, which indicates that the release mechanism is influenced by the diffusion rate and swelling of the polymer.^58,59^ In the Peppas-Sahlin model, constant *k*_1_ is much higher than *k*_2_, meaning Fickian diffusion is the predominant mechanism. Interestingly, Free DICLO in 1% and 0.5% differs the most from the rest of the samples, primarily the result for Peppas–Sahlin, where *k*_1_ and *k*_2_ are almost no different from each other. Analysis *via* the Peppas–Sahlin model yielded large, nearly identical kinetic constants (*k*_1_ and *k*_2_).^19,46,60^ This lack of parameter identifiability prevents the reliable separation of the diffusion (*k*_1_) and relaxation (*k*_2_) contributions. Consequently, a confident mechanistic attribution based on the individual values of *k*_1_and *k*_2_ could not be established from this dataset.

## 4. Discussion

The goal of this study was to identify changes in poloxamer gelation characteristics and their corresponding effects on drug delivery properties upon the addition of diclofenac sodium salt and paracetamol to poloxamer-based hydrogel matrices. The primary objective was to evaluate the controlled release behavior and functional performance of the developed hydrogel system, with a focus on release characteristics and biological evaluation rather than extensive structural characterization. Techniques such as FTIR, SEM, and XRD can provide important information regarding the physicochemical properties of hydrogels; however, these characterizations are planned for future work to complement the functional data presented here.

The previous work on poloxamer hydrogels was selected based on their gelation temperature, physicochemical properties, and biological impact on cells.^47,61^ Differences in gelation temperatures of poloxamer systems with different compositions followed a trend that roughly matched previously observed trends in ion-specific effects on macromolecular solubility. In our study, the amount of drug released increased with concentration and preparation method. Although variations in the gelation behavior of poloxamer systems could reflect changes in the properties of micelles forming the gel structure, we observed little evidence of a corresponding effect on drug delivery. The formulation of diclofenac sodium salt at 0.5% and paracetamol at 0.5% as a hydrogel-loaded matrix successfully produced a controlled-release formulation that improved *in vitro* release. The poloxamer-based material was made as a good final dosage form with acceptable appearance, pH, and viscosity.^56,62,63^

It is known that increasing the copolymer molar mass and PPO content reduces the minimum gelation concentration, while increasing the hydrophobic PPO block content lowers the critical micellar temperature.^7,57^ Poloxamer 188 (PEO_153_PPO_29_PEO_153_), for example, exhibits a gelation temperature that is often higher than that of Synperonic F108 (PEO_265_PPO_50_PEO_265_). Both Synperonic F108 and Kolliphor P407 contain PPO blocks of similar lengths, 50 and 56 units, respectively. Diclofenac sodium is an ionic salt with a strong tendency to interact with the hydrophilic PEO blocks. This small difference occurs because the portions of PEO blocks that emerge at the PPO boundary tend to form, for topological reasons, a compact corona around it with limited or no interpenetration of water molecules, resulting in a low contrast in electron density with the effective PPO core.^61,64^

The nature, composition, and concentration of poloxamers are the most critical factors in defining the rate of drug release from an *in situ* gelling matrix.^1^ Hydrophobic gel matrices exhibit compact micellar arrangements, leading to slow diffusion and erosion. Recent advances have addressed the inherent limitations of poloxamer hydrogels, including weak mechanical properties and rapid gel erosion, through chemical crosslinking strategies that extend drug release profiles up to 70 days while maintaining biocompatibility.^65,66^ Depending on the intended clinical use, gel properties can be adjusted through physical blending or chemical crosslinking with additive materials to slow release and enhance residence time at the application site.

Further discussion on kinetic modeling confirms that the choice of mathematical models for analyzing release from swelling matrices must align with the polymer's physical response to hydration.^44,67^ The observed complexity in fitting the Peppas–Sahlin model, indicated by parameter non-identifiability (*k*_1_ ≈ *k*_2_), suggests that the diffusion and relaxation contributions are highly coupled, characteristic of anomalous transport behavior in polymeric networks. The tendency of the release to deviate from simple Fickian diffusion (as suggested by the Korsmeyer–Peppas exponent *n* 0.85) underscores the dominant roles of matrix swelling and polymer chain relaxation in controlled drug release from these stimuli-responsive hydrogels.^68,69^ The drug release kinetics were best analyzed using the Peppas–Sahlin model, suggesting that release is governed by an anomalous transport mechanism or predominantly Fickian diffusion, rather than simple Fickian or Case II transport alone.

Release behavior is intrinsically linked to the thermoresponsive nature of poloxamers. Systems maintained in the gel state exhibited significantly restricted drug release compared to the liquid state at 4 °C.^70^ The differential release rates observed between paracetamol and diclofenac formulations highlight that the specific drug–polymer interaction and its influence on micelle packing dictate the final kinetic profile.^42,71^ Hydrophobic small molecules can be encapsulated within poloxamer matrices; however, these additives often negatively affect the rheological properties and reduce the gelation temperatures of the hydrogels, potentially limiting their clinical use.^72^ Studies employing differential scanning calorimetry, rheology, and small-angle X-ray scattering have demonstrated that the addition of small molecules lowers thermal transition temperatures and increases micelle size, providing a fundamental understanding for tuning the mechanical and structural properties of drug-loaded formulations.^72^

The overall biocompatibility, supported by the Vero cell viability data, aligns with the recognized benefits of poloxamer hydrogels, which facilitate controlled release while promoting a permissive microenvironment for cell attachment and growth.^43^ Critically, *in vitro* cytotoxicity assessments using the resazurin assay on Vero cells indicated that extracts loaded with paracetamol or diclofenac presented high cell viability, generally supporting proliferation at longer incubation times, which is consistent with the capacity of hydrogels to offer a permissive microenvironment.^7,73^ Recent studies on poloxamer-based binary hydrogels have demonstrated reduced cytotoxicity on fibroblasts and hepatocytes, with prolonged analgesic effects pointing to poloxamer-based hydrogels as potential treatments for acute pain *via* subcutaneous injection.^74^

Injectable hydrogels have emerged as promising options for osteoarthritis treatment due to their ability to deliver bioactive molecules directly to the affected joint, thereby enhancing local effectiveness and reducing systemic side effects.^75,76^ Recent clinical and preclinical studies have validated the use of anti-inflammatory agents in thermosensitive hydrogels for intra-articular applications, showing regenerated cartilage profiles and superior anti-inflammatory effects in osteoarthritis models.^77^ Thermoresponsive hyalomer intra-articular hydrogels containing poloxamer 407 and diclofenac have demonstrated the highest anti-nociceptive and anti-edematous effects, with radiography and histopathology revealing regenerated cartilage profiles in treated groups.^78^ These findings support the potential application of the developed formulations for localized drug delivery in meniscus regeneration and osteoarthritis treatment.

Regarding storage conditions and stability, the present study employed storage conditions that do not interfere with the properties of the tested hydrogels. The literature confirms that refrigerated storage at 4 °C in sealed, sterile containers is recommended for poloxamer-based hydrogels to maintain their physicochemical properties and ensure suitability for clinical use.^38,79^ The main objective of this work was to explore the development of personalized injectable hydrogels designed for individual patient needs, with drug release studies and formulation preparation carried out shortly before use. Therefore, long-term stability studies were not included in the current manuscript. Nevertheless, rheological analyses confirmed that the hydrogel maintains structural integrity and injectability during typical handling.^7^ The formulation retained key properties under refrigerated storage at 4 °C, and drug-loaded hydrogels demonstrated stability with controlled degradation and sustained drug release over the 28-day study period. Injectable hydrogels, serving as multifunctional biomaterials, exhibit exceptional shear-thinning behavior, tunable mechanical properties, and biocompatibility, owing to their dynamic porous network structure and biomimetic microenvironment.^20,59,80^ For clinical translation, the fluid nature of the selected formulations at room temperature enables straightforward sterilization by filtration while in their liquid state, a critical requirement for commercial use. Previous studies on thermoresponsive hyalomer formulations have demonstrated maintained percentage drug release and drug content after 3-month storage, supporting the feasibility of poloxamer-based systems for clinical applications when appropriate storage conditions are employed.^47,81^

The current challenges associated with clinical translation of injectable hydrogels include achieving optimal mechanical properties, ensuring long-term stability, and meeting regulatory requirements.^82^ Strategies to overcome these hurdles include physically blending with other polymers, chemically crosslinking, and incorporating drug-loaded particles to create multiple rate-limiting release barriers. Future research directions emphasize enhancing clinical applicability and overcoming current technological and clinical barriers to promote widespread use in precision medicine and personalized therapies.

Overall, the results suggest these compositions possess desirable characteristics, including established biocompatibility and temperature-dependent controlled release, making them promising candidates for future local drug delivery applications.^83,84^ The poloxamer concentration and composition significantly influence their biomedical applications, with triblock copolymer systems showing promising features for surgical applications, such as favorable sol–gel transition kinetics and biocompatibility, thus suggesting potential applications in osteoarticular regeneration.

## 5. Conclusions

Poloxamer-based injectable hydrogels effectively deliver anti-inflammatory agents with controlled release behavior, enhancing targeted treatment while minimizing systemic exposure. The formulations developed in this study demonstrated appropriate sol–gel transition near body temperature, stable pH, and osmolarity suitable for biomedical use. Drug release profiles showed controlled, sustained release of both diclofenac sodium and paracetamol over time, with mathematical modeling indicating that diffusion-based mechanisms predominated in drug release from the hydrogel matrix.

The cytotoxicity assessments confirmed high biocompatibility of the developed formulations, with cell viability remaining above acceptable thresholds for most conditions tested. The paracetamol-loaded formulations exhibited superior biocompatibility compared to diclofenac-loaded systems at early time points, while both formulations supported cell proliferation at longer incubation times.

The kinetic analysis revealed that the Peppas–Sahlin model best described the release behavior, indicating anomalous transport mechanisms where both diffusion and polymer relaxation contribute to drug release. The temperature-dependent release profiles reflect the fundamental relationship between poloxamer structure and drug diffusion, with the gel state at physiological temperatures providing more controlled release than the liquid state.

These hydrogels represent a versatile platform for localized drug delivery in regenerative medicine and orthopedic applications, supporting improved therapeutic outcomes and reduced side effects. The personalized approach to formulation preparation, with drug loading performed shortly before use, aligns with current trends in precision medicine and patient-specific therapies. Future work will include comprehensive structural characterization, extended stability studies, and *in vivo* evaluation to further validate the clinical potential of these formulations for meniscus regeneration and osteoarthritis treatment.

## Author contributions

All authors (M. T., A. G., J. S.-W., P. S., A. B.) have participated actively in the conception and design of the article. M. T. – conceptualization, data curation, investigation, methodology, original draft; A. G., P. S. – data curation, methodology; J. S.-W., A. B., P. S. – supervision, editing (review and editing). All authors have read and agreed to the published version of the manuscript.

## Conflicts of interest

The authors declare no conflicts of interest.

## Data Availability

The data presented in this study are available at the repository link provided. See https://box.pionier.net.pl/d/9bbc61463d0a4180b533/. Supplementary information (SI) is available. See DOI: https://doi.org/10.1039/d6ra02085b.
